# Sea-Bathing

**Published:** 1871-07

**Authors:** 


### SEA-BATHING,

As practiced at our fashionable watering places, as a means of health, is one of the greatest absurdities of modern times; it kills more than it cures; more than that it is a toil, a labor, an indecency, especially for ladies; the trouble of dressing and undressing, the ride or walk to the beach under a burning sun, along roads which suffocate with their dust, and the thousand and one discomforts, annoyances, and troubles inseparable from the practice, are great bores. That some will be certainly drowned there can be no dispute, and for their benefit we state the best means for their friends to take to bring them back to life.

Unless in cold weather, don't waste time in carrying the body to a house; but right on the spot, within a rod of the water's edge, remove all clothing, as far down as the navel; place it on its face over a bundle of straw or other soft substance, so that the stomach shall rest upon it; press spasmodically with both hands on the back, so as to force all the water possible out of the lungs through the throat and mouth, outward; the next instant place the body face upwards, something being under the small of the back, and draw out the tongue; then let another person get astride of the body, and with both hands, one on each side of the ribs, press them upward towards the mouth, as if you wanted to force the water out of the lungs, through the mouth, for a few seconds; then withdraw the hands as suddenly as possible, put them on the ribs again, and proceed as before; the object being to push out the water with one operation, and by the other to allow the air to rush into the lungs to supply its place. Keep on working in this way until you see some signs of life, even if you have to work for two or three hours. As soon as breathing is restored, convey to a bed if possible, and wrap up the naked body, from head to foot, in woolen blankets, in a warm room, with a plenty of fresh air; meanwhile give a drink of some hot liquid every five minutes, for the first hour or two, allowing the body the most perfect rest possible all the time, and besides promote composure of mind with as much encouragement as can be conscientiously given.

Life is lost by drowning, simply by the water getting into the lungs and filling them up, thus preventing any air from getting into them; and a man dies on the same principle as if he was hung, or smothered with a pillow, or put in an exhausted air receiver; all the operations above named, if studied, will be seen to promote the getting of the water out of the lungs; the next point is to give natural warmth, which is antagonistic to death. Taking the whole matter into consideration, it would seem to be a better plan not to indulge in sea-bathing, and thus prevent all this ado.

